# Loudness and Sound Category: Their Distinct Roles in Shaping Perceptual and Physiological Responses to Soundscapes

**DOI:** 10.1523/ENEURO.0146-25.2025

**Published:** 2025-09-18

**Authors:** Mercede Erfanian, Tin Oberman, Maria Chait, Jian Kang

**Affiliations:** Institute for Environmental Design & Engineering, University College London, London WC1H 0NN, United Kingdom; ^2^Ear Institute, University College London, London WC1X 8EE, United Kingdom

**Keywords:** eventfulness, pleasantness, SCR amplitude, SCR rise-time, skin conductance response (SCR), soundscape

## Abstract

When compared with nature sounds, exposure to mechanical sounds evokes higher levels of perceptual and physiological arousal, prompting the recruitment of attentional and physiological resources to elicit adaptive responses. However, it is unclear whether these attributes are solely related to the sound intensity of mechanical sounds, since in most real-world scenarios, mechanical sounds are present at high intensities or if other acoustic or semantic factors are also at play. We measured the skin conductance response (SCR), reflecting sympathetic nervous system activity as well as the pleasantness and eventfulness of the soundscape across two passive and active listening tasks in healthy subjects (*N* = 25; 14 females, 11 males). The auditory stimuli were divided into two categories, nature and mechanical sounds, and were manipulated to vary in three perceived loudness levels. As expected, we found that the sound category influenced perceived soundscape pleasantness and eventfulness. SCR was analyzed by taking the mean level across the stimulus epoch and also by quantifying its dynamics. We found that mean SCR was modulated by loudness only. SCR rise-time (a measure of the time it takes the skin response to increase from the baseline to its maximum value) correlated significantly with soundscape pleasantness and eventfulness for nature and mechanical sounds. This study highlights the importance of considering both the loudness level and sound category in evaluating the perceptual soundscape, and it identifies SCR as a valuable tool for such assessments.

## Significance Statement

While loud mechanical sounds are often deemed more unpleasant than nature sounds, it remains unclear whether this response is due to loudness itself or the origin of the sound. This study disentangles these effects by using skin conductance responses, a window into the body's automatic, unconscious reactions, to reveal that loudness is a key driver of physiological arousal. However, the listener's subjective perception of how pleasant or eventful a soundscape feels also significantly shapes this response. These findings highlight the dynamic relationship between physical sound properties and perceptual experience in shaping our reactions to everyday acoustic environments.

## Introduction

“Soundscape” is defined as an acoustic environment perceived, experienced, and/or understood by a person or people, in context (ISO 12913-1, 2014). It is proposed to be composed of two main perceptual attributes, pleasantness and eventfulness. These attributes are considered to be distinct from the intrinsic physical characteristics of the acoustic environment and function as evaluative metrics for auditory quality (ISO 12913-3, 2019; Erfanian et al., 2021). Pleasantness encapsulates the emotional resonance and affective magnitude of auditory perception, while eventfulness encompasses the perceptual intensity and dynamic variability of the auditory experience (Erfanian et al., 2019, 2021; Kang et al., 2019).

Considerable evidence suggests that particular acoustic properties, known as primary factors, may influence the soundscape (McDermott, 2012). These factors may include the spectral content of sounds, which contain substantial concentrations of energy in varying frequency ranges (Patchett, 1979; Kumar et al., 2008) or specific temporal modulations (Kumar et al., 2008; Arnal et al., 2019). The composition of these acoustic properties may be inherent to different sound sources (e.g., traffic noise and a public park) in the context of urban areas which gives rise to the variance in the appraisal of those sound sources, making them pleasant or unpleasant (Bradley et al., 2000a,b; Gomez and Danuser, 2004; Hume and Ahtamad, 2013; Medvedev et al., 2015). Mounting evidence suggests that nature sounds like ocean waves characterized by certain acoustic properties (high energy in low frequencies), contribute to the psychological and physiological benefits [through increased activity of the parasympathetic nervous system (PSNS); Bradley et al., 2000a; Alvarsson et al., 2010; Medvedev et al., 2015; Hedblom et al., 2019; Li and Kang, 2019; Buxton et al., 2021]. In contrast, mechanical sounds, like sirens with high energy in high frequencies, induce unpleasantness, accompanied by an increase in the sympathetic nervous system (SNS) activity which can be quantified through physiological indicators such as skin conductance response (SCR; Medvedev et al., 2015; Li and Kang, 2019). Although spectral content and temporal modulations shape perceptual responses, the dominant and most widely studied primary factor that determines unpleasantness is the loudness level (Skagerstrand et al., 2017; Mitchell et al., 2021; Carraturo et al., 2024; Oszczapinska et al., 2024).

Despite the availability of in situ studies (Axelsson et al., 2010; Jo and Jeon, 2020; Erfanian et al., 2021; Tarlao et al., 2022; Aletta and Torresin, 2023), there remains a dearth of evidence regarding the nature of unpleasantness assessments in response to mechanical sounds. This issue emerges because, in most urban real-world scenarios, mechanical sounds are usually associated with higher intensity than nature sounds. In addition, previous laboratory-based research presented mechanical sounds as louder relative to nature sounds, eliciting stronger perceptual and physiological representations (Gomez and Danuser, 2004; Hume and Ahtamad, 2013; Medvedev et al., 2015; Li and Kang, 2019; Tavano and Poeppel, 2019). Therefore, it remains inconclusive whether overall loudness alone influences these perceptual and physiological attributes or if the combination of overall loudness with other unique acoustic features accounts for the observed differences between nature and mechanical sounds.

SCR is a phasic, stimulus-locked change in the electric conductivity of the skin. It has been widely used to measure physiological activity in response to sounds (Schweiger and Maltzman, 1985; Bradley et al., 2000a,b; Gomez and Danuser, 2004; Dawson et al., 2007; Benedek and Kaernbach, 2010; Lang and Bradley, 2010; Boucsein, 2012; Medvedev et al., 2015;Greco et al., 2016; Gatti et al., 2018; Li and Kang, 2019). Under consistent environmental conditions (e.g., room temperature), the amplitude of the sudomotor nerve burst, driven by the intensity of stimuli such as sound [sound pressure level (SPL)], is linearly related to the number of recruited sweat glands and the corresponding SCR (Wallin, 1981; Benedek and Kaernbach, 2010; Bach, 2014). Sweat glands are predominantly innervated by sympathetic cholinergic fibers originating from the sympathetic chain (Shields et al., 1987). Additionally, the activity of sweat glands is strongly modulated by the limbic system, which is involved in affective sound processing (Fruhholz and Grandjean, 2013; Fruhholz et al., 2016). Beyond SCR amplitude, additional indices can be leveraged in experimental paradigms including SCR rise-time. The SCR rise-time is the temporal interval between the onset of the response (SCR initiation) and its peak amplitude, during which the current rises from 10 to 90% of its final value (Dawson et al., 2007; Boucsein, 2012). The rise-time of SCR offers insights into the speed of the physiological response to a stimulus (Venables and Christie, 1980; Dawson et al., 2007; Jindrová et al., 2020). A shorter rise-time indicates a more rapid physiological response, which can be seen in situations where a stimulus elicits a strong emotional response. A longer rise-time may be indicative of a more gradual or muted physiological response, which may occur in response to a weaker or less emotionally salient stimulus. Taken together, this evidence suggests that SCR is a useful method for quantifying the physiological basis of soundscape properties.

Our study aims to address two primary objectives: the first is to determine whether loudness, a percept driven by the SPL (in decibel) as well as frequency (in hertz), contributes to variance in the pleasantness and eventfulness and the underlying SCR. The second aim is to examine the effects of two distinct sound categories, nature and mechanical, on pleasantness and eventfulness, and their associated SCR. This classification of sounds is supported by previous research on sound taxonomy (Salamon et al., 2014; Bones et al., 2018).

To address these objectives, we measure the pleasantness, eventfulness, and SCR in response to complex, single-sourced nature and mechanical sound scenarios presented at three loudness levels of low (10 sones), medium (20 sones), and loud (30 sones) over a period of ∼15 s in two separate listening tasks (passive and active) in 25 healthy participants. We expect that loudness would lead to a decrease in perceptual pleasantness, whereas it would result in an increase in eventfulness and SCR. Furthermore, if overall intensity is the only inherent characteristic leading to differences between nature and mechanical sounds, we predict that nature and mechanical sounds at the same loudness level (e.g., 10 sones—low) derive similar subjective and objective responses.

## Materials and Methods

### Participants

Thirty-two paid participants took part in this study (17 females, 15 males; age mean, 28.3 ± 10.61; age range, 18–45). They reported no hearing/auditory difficulties/impairment and no neurological or relevant health dysfunctions. The same cohort of participants engaged in both the initial passive listening task and the subsequent active listening task. All participants were briefed on the experimental protocol, provided written informed consent, and were remunerated for their participation. Participants were recruited from UCL at Here East. The experimental procedures were approved by the Ethics Committee of University College London.

We excluded one participant due to inattentiveness during the passive listening task. Four participants were excluded due to SCR “lability” (nonresponders). This refers to subjects who show spontaneous fluctuations of SCR in the absence of specific stimulation [nonspecific SCR (NS-SCR) or slow SCR habituation (<0.01 μs); Prokasy and Raskin, 1973]. According to Venables and Mitchell (1996), ∼25% of the normal population is SCR labile. The subjects’ lability was determined by visually tracking the real-time data during the experiment. Two further participants with >50% bad trials (movement artifacts and electrode artifacts) were excluded during the data preprocessing.

The final analyzed data therefore are based on 25 retained participants (14 females, 11 males; age mean, 27.44 ± 9.76; age range, 18–45).

### Auditory stimuli

The auditory stimuli consisted of 15-s-long prerecorded single-sourced acoustic “scenes” downloaded from “freesound.org” and “sound-effects.bbcrewind.co.uk” in two categories of “mechanical“ and “nature” sounds. In each category, we had three distinctive sound scenarios: highway, jackhammer, and chainsaw for mechanical sounds and waterfall, bird chirping, and crickets for nature sounds. Of each scenario, four different exemplars were used. These stimuli were selected based on the International Affective Digitized Sounds database “list”—expanded version of the second edition (IADS - 2; Lang and Bradley, 2007). The IADS-E auditory stimuli (*N* = 935) are classified by their semantic categories, and the dataset includes the arousal and valence ratings collected from Japanese students (*N* = 207; Yang et al., 2018). Since the auditory stimuli in the IADS-E were short (1.5 s), we selected task stimuli with the same labels (e.g., jackhammer in the mechanical sound category) but with longer durations from other sources. Although these sounds shared the same semantic labels as those in the IADS-E (e.g., jackhammer), they cannot be assumed to match its normative arousal and valence ratings. Instead, the IADS-E ratings were only used to guide the selection of sound categories and exemplars expected to span the affective space.

While the selected stimuli were intended to represent a range of affective values within each category, we acknowledge that the six scenarios (three per category, each with four exemplars) constitute a relatively narrow and selective sample of possible nature and mechanical sounds. As such, this specificity may limit the generalizability of our findings to other soundscapes within these broader categories. Our intention was not to exhaustively sample the category space but rather to use a controlled subset of representative sounds to examine affective and physiological responses across distinct semantic and emotional profiles. To select auditory stimuli labels in both natural and mechanical sound categories, we determined the 0.25 (Q1), 0.5 (Q2), and 0.75 (Q3) quantiles based on the arousal (nature ranging from 2.58 to 7.83; mechanical ranging from 2.18 to 8.58) and the valence scale (nature ranging from 1.82 to 8.25; mechanical ranging from 1.27 to 7.09). Subsequently, the sound exemplars whose mean arousal and valence values matched with Q1, Q2, and Q3 were methodically selected, including a waterfall (arousal 3.9, valence 6.6), highway (arousal 3.8, valence 5.6), crickets (arousal 5.2, valence 5), a chainsaw (arousal 5.4, valence 4.2), bird chirping (arousal 6.5, valence 3.7), and a jackhammer (arousal 7, valence 2.8), representing nature and mechanical sounds, respectively.

**Figure 1.**
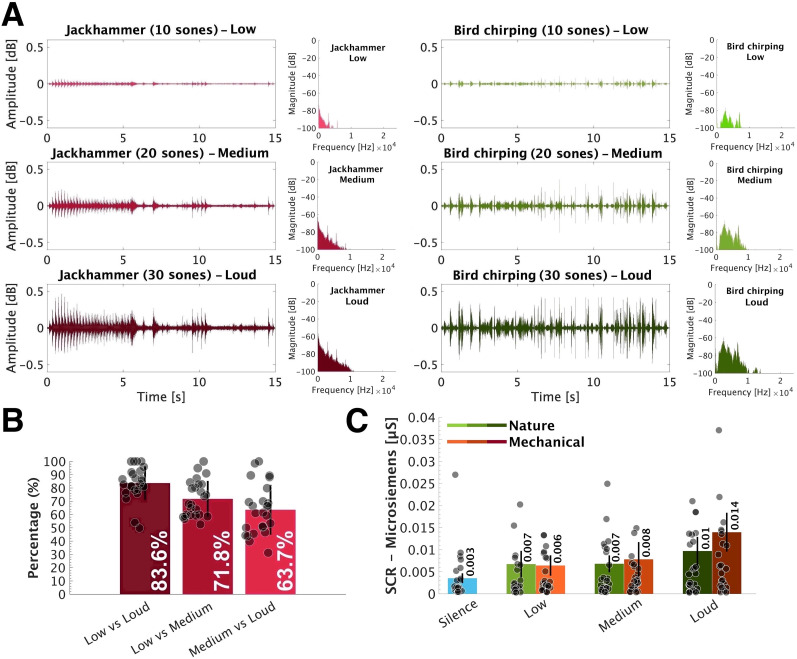
***A***, Exemplars of mechanical (Jackhammer) and nature (Bird chirping) sounds at three loudness levels: low (10 sones), medium (20 sones), and loud (30 sones) in the time and frequency domains. B, Behavioral performance of loudness discrimination task. Error bars (SD) illustrate the variability in performance with circle illustrating individual data. C, The averaged SCR across all trials and all participants (N = 25) in nature (light green to dark green) and mechanical (orange to brown) and blue representing the silence condition. Error bars indicate ±1 SEM. Gray circles indicate individual data. SCR measures were significantly modulated by increasing the loudness of nature and mechanical sounds (*** < 0.001; ** < 0.01; * < 0.05). Figure contributions: Mercede Erfanian performed the experiment and analyzed the data.

The sounds were normalized at three loudness levels, ∼10 sones (low), 20 sones (medium), and 30 sones (loud), making a total of 72 trials (Fig. 1*A*). The stimuli were normalized by the ArtemiS SUITE HEAD acoustics software version 10.7 and measured in the laboratory by Head acoustics SQobold with a BHS II headset. None of the auditory stimuli were above the hazardous threshold of 85 dB SPL (Costa et al., 2022).

Auditory stimuli were delivered to the participants through 12 coaxial loudspeakers (Genelec 8030A) placed to follow an imagined sphere (*r* = 1.5 m) in three rows around a participant on the floor, 1.5 m height and 3.0 m height. The principle used to deliver two-channel stereo recordings to the speaker array was based on routing channel one to six speakers on the left side and channel two to six speakers on the right side where the four middle speakers were assigned in a way to keep the balance of two speakers per channel on each height. Stimulus presentation was controlled with Psychtoolbox [Psychophysics Toolbox version 3 (Brainard, 1997) on MATLAB (version 2019a; MATLAB, 2019)]. The interstimulus interval varied between 30 and 60 s (randomly; in steps of 5 s).

### Loudness discrimination task

Prior to the main experiment, we conducted a short loudness discrimination task on participants to ensure the three loudness levels used were distinguishable. The participants were presented with one block which consisted of 12 pairs of stimuli selected from all three loudness levels. Of the 12 pairs, 3 pairs contained stimuli with the same loudness levels (low vs low, medium vs medium, and loud vs loud), 3 pairs of low vs medium, 3 pairs of low vs loud, and 3 pairs of medium vs loud. Participants were instructed to press a designated key on a provided keyboard to indicate the louder sound and to not react when sounds were presented at the same loudness level. The trials were randomized across participants (Fig. 1*B*).

### Procedure

**Figure 2.**
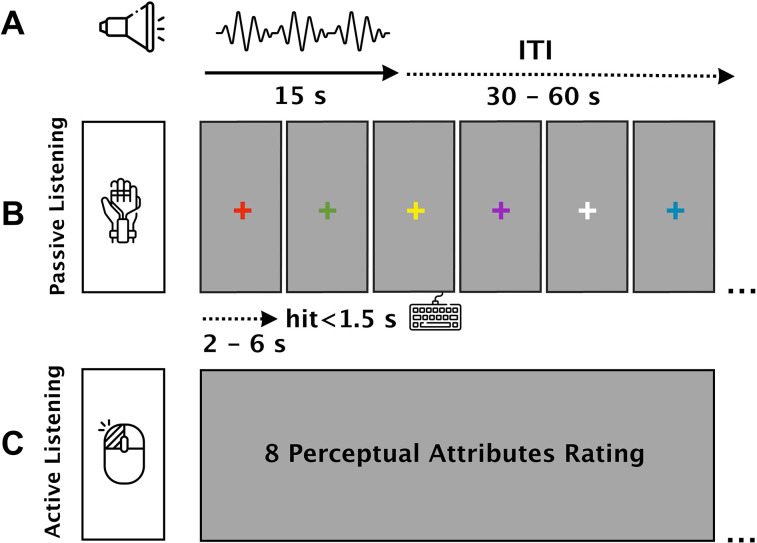
Task structure and timing across conditions. ***A***, Schematic of auditory stimulus timing: each sound lasted 15 s and was followed by a variable intertrial interval (ITI) ranging from 30 to 60 s. ***B***, A passive listening task, in which participants listened to each auditory stimulus without providing ratings. SCRs were recorded continuously to index autonomic arousal during the passive listening task. To promote attention during this task, an incidental detection task was embedded: participants were instructed to press a designated key as soon as the fixation cross on the screen changed color. A “hit” response was defined as a keypress within 1.5 s of the color change onset. The color change occurred at random intervals ranging from 2 to 6 s. ***C***, An active listening task, during which participants were instructed to attend to the same auditory stimuli and rate each on eight perceptual attributes. No physiological data were recorded during this task. Figure contributions: Mercede Erfanian performed the experiment and analyzed the data.

The study consisted of two separate parts of “passive” and “active” listening tasks, followed by debriefing (Fig. 2). To control for undesirable environmental factors such as noise interference and temperature and to ensure that the SCR fluctuations were induced by the sound stimuli, we included six incidental trials [silence conditions (15 s)] in which no stimulus was presented to the participants. The silence conditions were spread randomly across blocks with one incidental trial in each block.

### Passive listening task (SCR recorded)

The participants sat in front of a monitor [23.8 in. BenQ 2480 Full HD with the WQHD resolution (2,560 × 1,440 pixels)] in a dimly lit and acoustically shielded room. They were instructed to sit still and to continuously fixate on a changing color fixation cross (∼2 × 2 cm) presented in the center of the screen against a light gray background with a distance of ∼0.5 m while being presented with the sounds. To assess the level of participants’ attention to the stimuli, they were directed to engage in a task where they were required to promptly press a space bar, located on a provided keyboard, as soon as the color of the fixation cross changed (every ∼2–6 s). For an accurate recording of attention, a keypress was classified as a “hit” only if it occurred within a time frame of <1.5 s following the change of the fixation cross color. Auditory stimuli were presented randomly with interstimulus intervals of 30–60 s (Fig. 2*B*).

### SCR

The SCR was measured, using Empatica4 (CE Cert. No. 1876/MDD; 93/42/EEC Directive, Medical Device class 2a - FCC CFR 47 Part 15b) which is an ambulatory device, normally wrapped around the wrist. Its reliability is comparable with clinical devices in appropriate circumstances (McCarthy et al., 2016). Since palmar and plantar areas showed to have stronger SCR and the wrist sweat glands may be primarily thermoregulatory in function (Dawson et al., 2007; Boucsein, 2012), we attached the sensors of the device to the index and middle fingers of the participants’ nondominant hands. The participants were instructed to minimize their movement during the experiment. SCR real-time data (via SCR sensor) and participants’ movement (via three-axis accelerometer sensor) were continuously monitored and recorded by the Empatica4-manager software (version 2.0.1.5023) at a sampling rate of 4 Hz. Each block started with 120 s SCR baseline measure to obtain a stable baseline signal, and after each block, the data were automatically transferred to the Empatica4 secure cloud platform which was exported from the cloud for analysis.

To minimize fatigue, the participants were given breaks of 2–3 min between each block and a long 5–10 min break between the passive and the active listening tasks. At the end of the passive task, the Empatica4 was detached from the participants’ fingers. The SCR was measured only during the passive listening task.

### Active listening task (perceptual attributes ratings; no SCR recording)

Subsequent to the recording of SCR during the passive listening task, an active listening task was conducted. The active listening task involved the random presentation of the same auditory sequence (15 s), with varying interstimulus intervals (30–60 s). Participants were instructed to rate each stimulus using eight perceptual attributes, ranging from 0 (min) to 100 (max) during the given intervals by pressing the left and right navigation keys to move the cursor along a slide bar. All perceptual attributes were presented simultaneously on the same page, with their order counterbalanced across trials for each participant. For each trial, participants were allotted a maximum of 25 s (∼3 s for each attribute) to register their responses. If no response was recorded within this time frame, the trial was automatically advanced to the subsequent trial. All eight perceptual attributes appeared during and after each sound stimulus for rating (Fig. 2*C*), and no SCR was recorded during this task.

### Perceptual attributes

The assessment of pleasantness and eventfulness was done by using an adapted version of ISO/TS, 12913-3:2019, based on the Swedish Soundscape Quality Protocol (SSQP; 41; ISO 12913-3, 2019). It includes a question “To what extent do you agree/disagree that the present sound is…?”. Using a continuous scale ranging from “0” as the minimum to “100” as the maximum, the participants evaluated the quality of the acoustic environment using eight adjectives: pleasant, chaotic, vibrant, uneventful, calm, annoying, eventful, and monotonous. Then, eight adjectives (PA) collapsed into two-dimensional coordinates plotted with continuous values between −100 and 100 for “pleasantness” on the *x*-axis and “eventfulness” on the *y*-axis. These dimensions were calculated as shown in Equations 1 and 2:

$$\mathrm{Pleasantness}\;(P)=\;\overset{8}{\underset{i=1}{\sum }}\;PA_{i}\;*\;\cos\;\theta_{i},$$

$$\mathrm{Eventfulness}\;(E)=\;\overset{8}{\underset{i=1}{\sum }}\;PA_{i}\;*\;\sin\;\theta_{i},$$

where PA_1_ is pleasant and *θ*_1_ is 0°; PA_2_ is vibrant and *θ*_2_ is 45°; PA_3_ is eventful and *θ*_3_ is 90°; PA_4_ is chaotic and *θ*_4_ is 135°; PA_5_ is annoying and *θ*_5_ is 180°; PA_6_ is monotonous and *θ*_6_ is 225°; PA_7_ is uneventful and *θ*_7_ is 270°; and PA_8_ is calm and *θ*_8_ is 315°.

The perceptual attributes as a measuring tool for the soundscape have been validated across several populations (Aletta et al., 2024).

### SCR preprocessing

The raw signal (recorded at 4 Hz and 0.001–100 µS) in response to auditory stimuli was preprocessed and analyzed by continuous decomposition analysis (CDA), using the Ledalab toolbox (v. 3.2.2) MATLAB (version 2019a; MATLAB, 2019). Prior to the CDA, data were visually inspected (blinded to the trial type), and movement artifacts were manually corrected by using spline interpolation. Then the CDA was carried out in four steps including deconvolution of SC data, estimation of tonic activity, estimation of phasic activity, and optimization.

These steps were initially performed for predefined parameters (*τ*_1_ = 1; *τ*_2_ = 3.75) to determine model fit. Subsequently, to enhance the goodness of the model, the parameters were optimized by reapplying these four steps.

1. Deconvolution of SC data: SC results from the convolution of sudomotor nerve bursts with the impulse response function (IRF), which describes the course of the impulse response over time. This process produces phasic and tonic components. We conducted deconvolution to reverse this process, allowing for the separation of phasic and tonic activity.
2. Estimation of tonic activity: Tonic electrodermal activity can occur without phasic activity (Boucsein, 2012), but the slow recovery of SCRs can obscure it. To estimate tonic activity, phasic response overlap was minimized by reducing its time constant, and then the time intervals between phasic impulses were used to estimate tonic activity. Deconvolution amplifies noise, so the tonic driver was smoothed using Gaussian convolution (*σ* = 0.2 s). Then peaks were detected by finding zero crossings in the first derivative, with significant peaks defined by a local maximum differing by *δ* ≥ 0.001 μS from adjacent minima. Nonoverlapping sections estimated the tonic driver, which was interpolated using a cubic spline along a 10 s grid size. Finally, tonic SC activity was reconstructed by convolving the tonic driver with the IRF.
3. Estimation of phasic activity: The phasic driver was obtained by subtracting the tonic driver from the total driver signal, resulting in a signal with a near-zero baseline and positive deflections, capturing the time-constrained nature of phasic activity.
4. Optimization: The IRF can vary based on individual skin characteristics. Thus, our initial parameters (*τ*_1_ and *τ*_2_) were optimized based on criteria that evaluate the model. First, it was important for the phasic driver to display clear, short bursts of activity that quickly returned to zero between these bursts. To check how clear these bursts were (indistinctness), the number of consecutive bursts that were above a set level (5% of the maximum value of the phasic driver) were counted and then divided by the sampling rate (4 Hz). This resulted in the duration of the bursts in seconds. Next, they were standardized by squaring, summing, and dividing by the total duration of the data, resulting in a measure in square seconds per second (*s*^2^/*s*). Higher values indicate longer bursts above the threshold. Second, the negative values in the phasic driver should be as low as possible. So, negativity was measured by calculating the RMS of the negative portions of the phasic driver. Finally, it created a criterion (*c* = indistinctiveness + negativity. *α*) that included both measures. The negativity measure was multiplied by a factor of *α* = 6 s^2^/(s µS) to guarantee both measures contribute similarly to the criterion (Benedek and Kaernbach, 2010). The parameters were optimized by minimizing the criterion (*c*). A low (*c*) score indicates a good model with a stable baseline and clear bursts of activity. The optimization was achieved using a gradient descent method (Snyman, 2005), which adjusted parameters to improve the criterion until no further significant improvements can be made [for more information; see Benedek and Kaernbach (2010)].

After the CDA, the CDA.SCR from each trial was epoched from the stimulus onset (*t*_0_) to 15 s postonset (corresponding to the stimulus duration). CDA.SCR is the Ledalab metric that refers to the average phasic driver within the response window but does not rely on traditional SCR amplitude calculations (µS). For each trial, baseline correction was applied by subtracting the mean SCR over the preonset interval (5 s preonset). Then data were averaged across all trials for each participant in each condition. The minimum threshold for SCR responses was set to 0.01 µS.

For time series analysis, after the CDA, the PhasicData (phasic activity) from each trial were epoched from 5 s prestimulus to 25 s postoffset. For each trial, as in the previous instance, baseline correction was implemented by subtracting the mean SCR measured during the preonset interval. Then the data for each participant in each block were normalized. To do this, the mean and standard deviation (SD) across all baseline samples (5 s preonset interval) in each block were calculated and used to *z*-score normalize all data points (all epochs, all conditions) in the block. For each participant, SCR was time domain averaged across all epochs of each condition to produce a single time series per condition.

### Statistical analysis

Statistical analysis was conducted in the MATLAB (version 2019a; MATLAB, 2019) and R statistical software (version 4.0.3). The *p*-value was a-priori set to *p* < 0.05 for all analyses. Two-way repeated–measures ANOVAs were conducted to analyze behavioral and physiological responses. Data were assessed for normality and sphericity, with Greenhouse–Geisser corrections applied when necessary. Post hoc comparisons following significant ANOVA results were performed using Games–Howell tests, with Bonferroni’s correction applied for multiple comparisons.

### Time series statistical analysis

A nonparametric bootstrap-based analysis (Efron and Tibshirani, 1994) was used to evaluate time interval differences in SCR across conditions (“silence,” “low,” “medium,” and “loud”) and (“nature” and “mechanical”). For each participant, time series differences between conditions were computed and subjected to bootstrap resampling (1,000 iterations with replacement). Statistical significance at each time point was determined by evaluating whether the proportion of bootstrap iterations exceeding (or falling below) zero surpassed the 95% confidence threshold (*p* < 0.05). Any significant differences observed during the prestimulus interval were attributed to noise. No difference was observed during the prestimulus interval.

## Results

### Incidental task performance is not modulated by loudness or sound category

For the incidental task (color change detection), performance (*d*′) was computed using hit (HR) and false alarm rates [*d*′ = *z* (HR) − *z* (false alarms)]. A space bar press was considered a hit if it occurred within 1.5 s following the change in color of the fixation cross. If HR or false alarms were at the ceiling, a standard correction was applied (Hautus, 1995). A parametric analysis (one-way ANOVA) with a Greenhouse–Geisser correction was performed to compare the conditions with factors of loudness level (10 sones “low,” 20 sones “mid,” and 30 sones “loud”). The performance measure revealed no difference between low (mean, 3.66; SEM, 0.04), mid (mean, 3.65; SEM, 0.04), and loud (mean, 3.63; SEM, 0.04) conditions (*F*_(2, 72)_ = 0.19; *p* = 0.83), indicating that performance on the incidental task was not affected by stimulus loudness.

Reaction times (RTs) were analyzed from each HR with a two-way repeated–measures ANOVA (factors of loudness and category) with a Greenhouse–Geisser correction. The results revealed no significant simple main effect of loudness levels (*F*_(1.83, 170.44)_ = 0.71; *p* = 0.48; *η*^2^ = 0.008) or sound categories (*F*_(1, 93)_ = 0.93; *p* = 0.34; *η*^2^ = 0.01) on RTs, and no significant interaction (*F*_(1.82, 169.63)_ = 0.01; *p* = 0.98; *η*^2^ < 0.001). These results suggest that participants succeeded in maintaining their attention during the task regardless of loudness levels or sound categories.

### SCR is modulated by loudness but not sound category

The SCR was computed as the average conductance value over the 15-s-long stimulus presentation and evaluated with two-way repeated–measures ANOVA with a Greenhouse–Geisser correction, with factors of the loudness level (“silence,” “low,” “medium,” and “loud”) and sound category (“nature” and “mechanical”). The results revealed a significant main effect of loudness, (*F*_(1.84,44.34) _= 7.33; *p* =0.002; *η*^2^ = 0.23), indicating that the induced SCR varies across loudness levels. In contrast, there was no main effect of sound category (*F*_(1,24) _= 1.79; *p* = 0.19; *η*^2^ = 0.07), confirming that SCR is strongly driven by the loudness levels rather than sound categories. Similarly, no interaction between loudness levels and sound categories was observed (*F*_(1.69,40.78)_ = 0.84; *p* = 0.42; *η*^2^ = 0.23; Fig. 1*C*).

Figure 1*C* plots the averaged SCR of all participants in the nature sound category and the mechanical sound category across loudness levels. A one-way ANOVA with a Greenhouse–Geisser correction was conducted with the loudness level (“silence,” “low,” “medium,” and “loud”) as factors across sound categories. To ensure that the elicited SCR was a result of stimulus presentation rather than NS-SCR, silence conditions were incorporated into the analysis. SCR measures yielded a main effect of loudness (*χ*^2^ = 4.92; *p*  = 0.02; *η*^2^ = 0.017) in nature sound condition. The post hoc test (Games–Howell test) demonstrated significant differences between loudness levels for silence (medium *p* = 0.005; loud *p* = 0.008), low (loud *p* = 0.003), medium (silence *p* = 0.005), and loud (silence *p* = 0.008; low *p* = 0.003). The differences between loudness levels were not significant including for silence (low *p* = 0.124), low (silence *p* = 0.124; medium *p* = 0.965), medium (low *p* = 0.965; loud *p* = 0.095), and loud (medium *p* = 0.095; across six pairwise comparisons). Similarly, in the mechanical sound category, the SCR measures showed a main effect of loudness (*χ*^2^ = 4.27; *p* = 0.02; *η*^2^ = 0.251). The post hoc test (Games–Howell test) demonstrated significant differences between some loudness levels for silence (medium *p* = 0.039; loud *p* = 0.01), low (loud *p* = 0.042), medium (silence *p* = 0.039), and loud (silence *p* = 0.01; low *p* = 0.042). On the other hand, some differences between loudness levels were not significant for silence (low *p* = 0.153), low (silence *p* = 0.153; medium *p* = 0.416), medium (low *p* = 0.416; loud *p* = 0.124), and loud (medium *p* = 0.124; across six pairwise comparisons).

Overall, the pattern is consistent with a monotonic increase in SCR with loudness.

**Figure 3.**
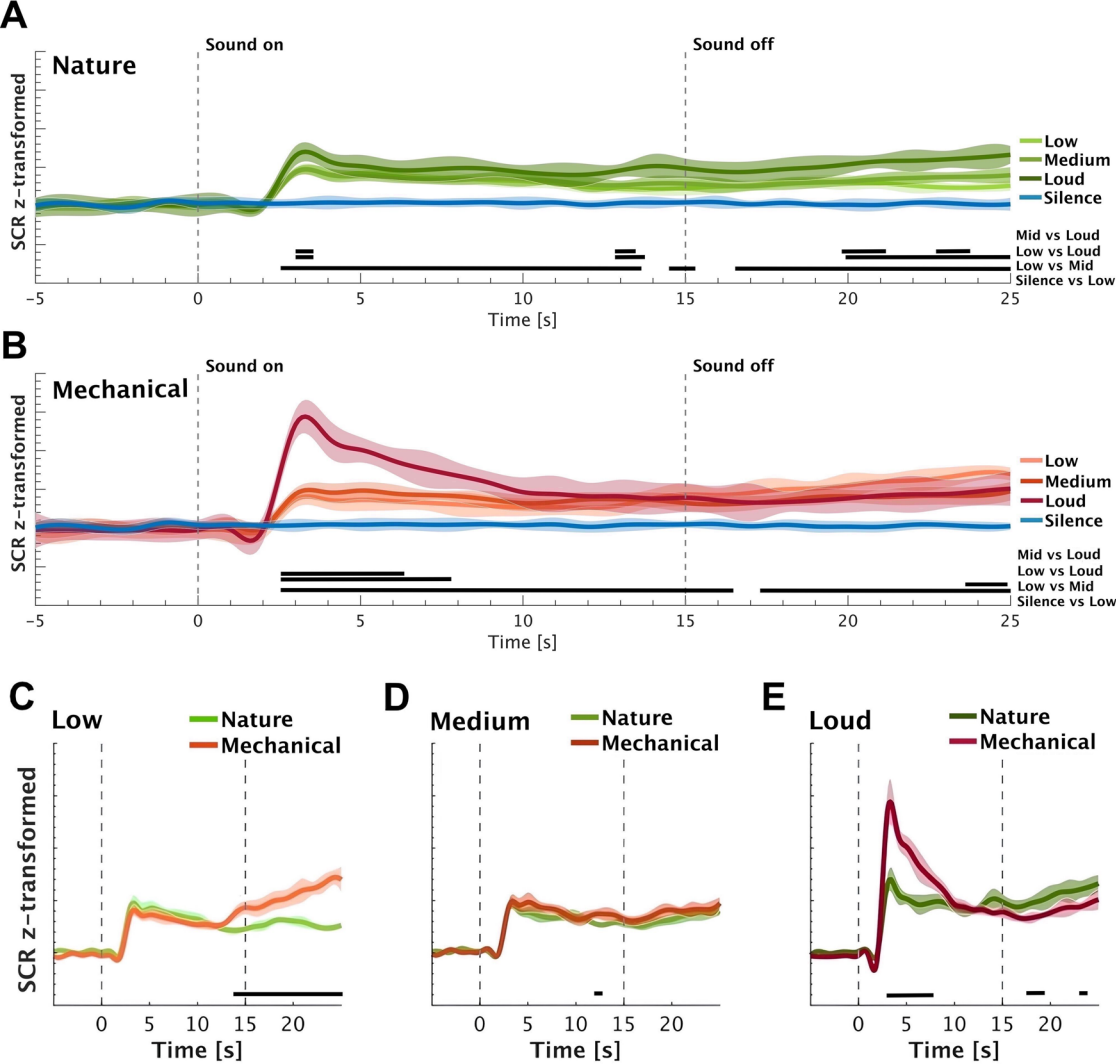
Comparison of the SCR across the conditions (“silence,” “low,” “medium,” and “loud”) in nature (***A***) and mechanical (***B***) sound categories. Panels ***C***–***E*** illustrate the SCR in response to nature and mechanical sounds under low, medium, and loud conditions, respectively. The shaded area shows ±1 SEM. The horizontal black lines represent the significant differences between conditions (*p* < 0.05). Figure contributions: Mercede Erfanian performed the experiment and analyzed the data.

The SCR time domain data are presented in Figure 3. All conditions exhibit a prototypical pattern of an abrupt decrease followed by a sharp increase in SCR at ∼2 s postonset, followed by a peak at ∼3 s postonset, with amplitude varying in proportion to the loudness level and then a slow decrease.

To identify time intervals in which a given pair of conditions exhibited SCR differences, a nonparametric statistical analysis was used (bootstrap-based analysis; demonstrated by the solid horizontal black lines in Fig. 3). Especially following onset, a clear gradation by loudness was observed for both stimulus types. Loud mechanical sounds evoked the strongest response. We proceeded to compare nature and mechanical sounds separately for each loudness level. A difference was observed between loud nature and mechanical sounds, unfolding ∼3 s after the stimulus onset and persisting until ∼7 s poststimulus presentation. This evidently suggests that loud mechanical sounds evoke stronger phasic activity relative to loud natural sounds, which may point to distinct characteristics of these sound categories. There was no significant difference between conditions at the “medium” loudness level. However, at the “low” loudness level, a notable divergence emerged late in the epoch, following sound offset: SCR to mechanical sounds exhibited an increase compared with those elicited by nature sounds. This pattern may suggest a delayed, slow-unfolding impact of mechanical sounds, even at low loudness levels. Given the incidental nature of this finding, however, we will not explore it further.

**Figure 4.**
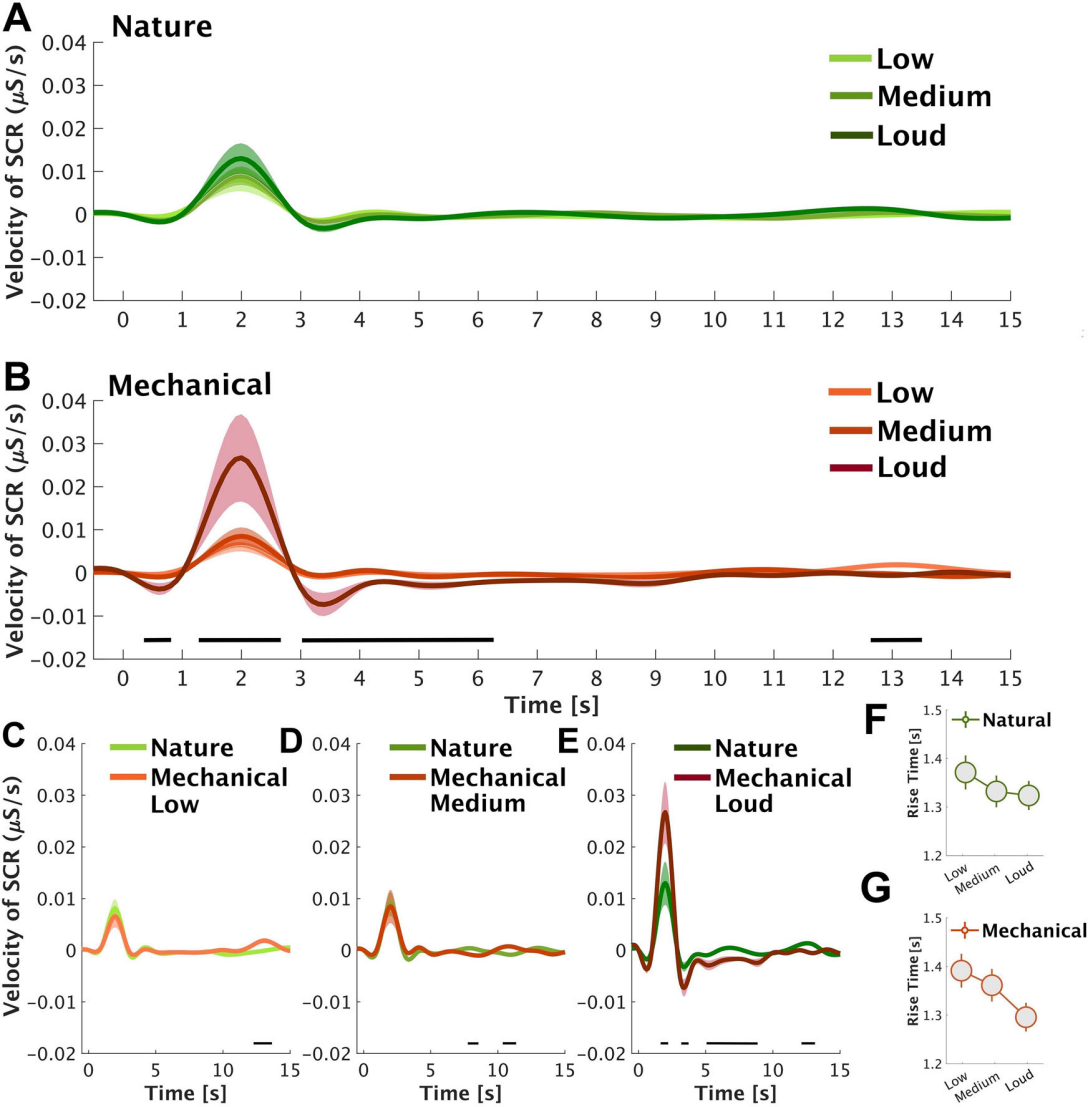
SCR velocity across loudness (“silence,” “low,” “medium,” and “loud”; ***A***, ***B***) and sound category (“nature” and “mechanical”; ***C***–***E***) conditions. The shaded area shows ±1 SEM. The black horizontal bars indicate time windows of significant differences. Panels ***F*** and ***G*** show SCR rise-time across loudness conditions. Figure contributions: Mercede Erfanian performed the experiment and analyzed the data.

To gain a more comprehensive understanding of SCRs, we further analyzed SCR velocity, which indicates the speed at which the SCR reaches its maximum following the sound onset. The velocity of SCR was quantified as the peak derivative during the SCR rise-time for each trial per participant, capturing trial-specific dynamics. These trial-level velocities were then averaged within each participant to obtain a participant-specific mean velocity profile for each condition (e.g., low nature, loud mechanical). Finally, these participant-level means were averaged across all participants within each condition to yield the group-level mean velocity, with SEM calculated to reflect interparticipant variability (Fig. 4). For nature sounds (Fig. 4*A*), the velocity responses show a small, transient peak ∼2 s poststimulus onset, followed by a return to the baseline. The magnitude of the response appears relatively consistent across conditions, with minimal difference in peak amplitude of loud nature sound. The data from mechanical sounds (Fig. 4*B*) reveal a peak at approximately the same time point but with a greater difference between conditions. The SCR velocity in response to loud mechanical sounds elicits the largest peak, while the low and medium mechanical sounds show more attenuated responses. Direct comparisons within loudness conditions reveal, consistently with the previous analysis, that high loudness mechanical sounds are associated with higher SCR velocity than nature sounds.

In addition, we analyzed the SCR rise-time. The SCR rise-time is identified as the point where the phasic component of the SC signal begins to increase following stimulus presentation, typically determined by a consistent upward deflection exceeding a predefined threshold (e.g., 0.01 µS). The peak is the maximum amplitude reached after onset, representing the highest conductance value before the response starts to decline. We tallied the SCR rise-time as the difference between the peak time and the SCR onset time, providing a measure of the duration at which the SCR reaches its maximum following initiation (Venables and Christie, 1980; Dawson et al., 2007; Boucsein, 2012). The SCR rise-time was computed for all conditions (“low,” “medium,” and “loud”) across nature and mechanical sounds in all subjects. We ran two one-way ANOVAs which revealed no difference between loudness levels in nature (*F*_(2,72)_ = 0.5; *p* = 0.6; *η*^2^ = 0.002) and mechanical (*F*_(2,72)_ = 1.55; *p* = 0.22; *η*^2^ = 0.005) sound categories. No post hoc analysis was conducted (Fig. 4*F*,*G*).

### Variance in pleasantness and eventfulness is driven by sound category

**Figure 5.**
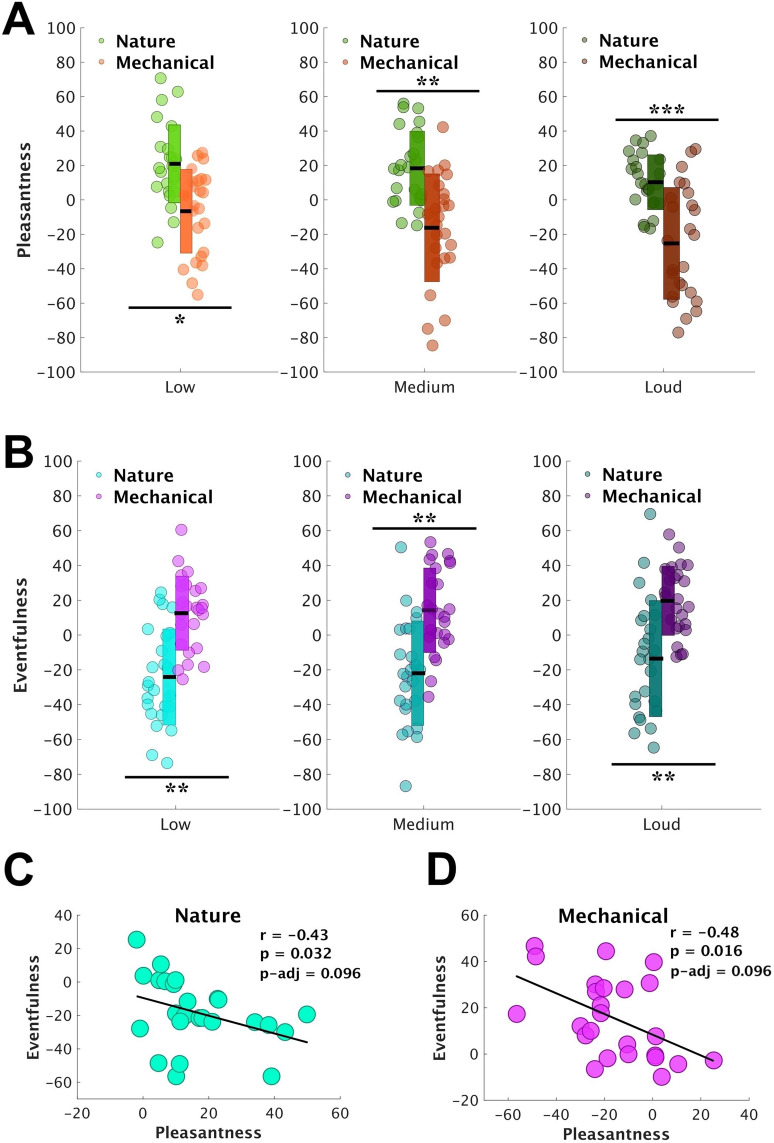
Soundscape pleasantness (***A***) and eventfulness (***B***) in response to nature and mechanical sounds across loudness levels (*N* = 25). The circles indicate the individual data. The black horizontal lines represent the mean. The thick vertical rectangle shows mean ± SD. Panels ***C*** and ***D*** show correlations (Spearman) between soundscape pleasantness and eventfulness across nature and mechanical sound categories (*** < 0.001; ** < 0.01; * < 0.05). Note: The correlations are exploratory and FDR-corrected *p*-values are reported in the figure. Figure contributions: Mercede Erfanian performed the experiment and analyzed the data.

Figure 5*A* plots averaged soundscape pleasantness across all participants (*N* = 25). We evaluated soundscape pleasantness using repeated-measures analysis (two-way repeated ANOVA) with a Greenhouse–Geisser correction with factors of loudness level (“low,” “medium,” and “loud”) and sound category (“nature” and “mechanical”). The analysis showed no main effect of loudness (*F*_(2,47.82) _= 4.32; *p* = 0.19; *η*^2^ = 0.15), indicating no difference in pleasantness between the loudness levels. The main effect of the sound category yielded a significant difference in soundscape pleasantness between nature and mechanical sounds (*F*_(1,24) _= 69.68; *p* < 0.001; *η*^2^ = 0.74), with nature sounds judged as significantly more pleasant. No significant interaction was found between loudness and sound category (*F*_(1.98,47.64) _= 0.53; *p *= 0.59; *η*^2^ = 0.02).

Figure 5*B* shows soundscape eventfulness across all participants (*N* = 25), evaluated with the same measure as soundscape pleasantness (two-way repeated–measures ANOVA with a Greenhouse–Geisser correction). Like the soundscape pleasantness, there was no main effect of loudness (*F*_(1.95,47.02) _= 1.76; *p* = 0.18; *η*^2^ = 0.07), whereas the main effect of the sound category was significant, revealing a difference in soundscape eventfulness between nature and mechanical sounds, with the latter being judged as significantly more “eventful” (*F*_(1,24) _= 46.22; *p* < 0.001; *η*^2^ = 0.66). No interaction was observed between loudness and sound category (*F*_(1.98,47.51) _= 0.09; *p* = 0.92; *η*^2^ = 0.004).

As has been reported previously (Erfanian et al., 2021; Mitchell et al., 2021), we observed a moderate negative correlation between pleasantness and eventfulness for both nature and mechanical sounds (Fig. 5*C*,*D*). However, after applying false discovery rate (FDR) correction, these correlations were no longer statistically significant. This confirms the theoretical framework underlying the ISO 12913-3 circumplex model (ISO 12913-3, 2019), in which pleasantness and eventfulness are intended to represent orthogonal perceptual dimensions. The absence of a robust correlation in our data thus suggests that these attributes may vary independently under controlled experimental conditions and that their inverse association in prior studies may be (1) due to lack of correction for multiple comparison or (2) context-dependent rather than intrinsic to the soundscape construct.

### Pleasantness and eventfulness do not correlate with SCR amplitude

**Figure 6.**
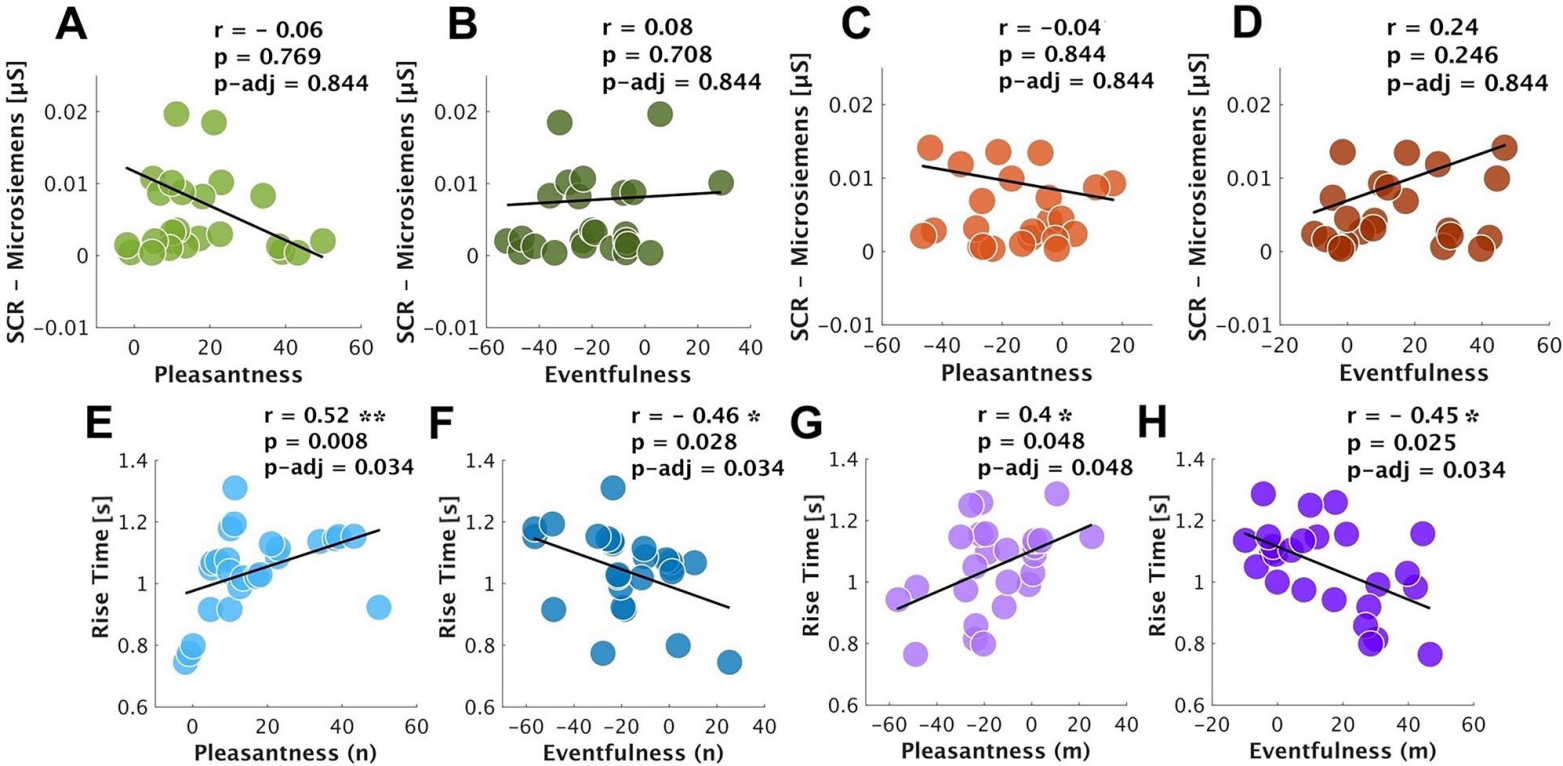
Correlation (Spearman) between soundscape pleasantness and eventfulness and the SCR amplitude (top) and the SCR rise-time (bottom) in seconds across nature (green and blue) and mechanical (orange and purple) sound categories (*N* = 25). Each circle represents individual data, and the diagonal line is the line of best fit, expressing the (degree of) relationships between the factors. Note: All correlations are exploratory and FDR-corrected *p*-values are reported in the figure (*** < 0.001; ** < 0.01; * < 0.05). Figure contributions: Mercede Erfanian performed the experiment and analyzed the data.

Spearman correlation was employed to investigate potential links between soundscape pleasantness and eventfulness and the SCR amplitude, separately for nature and mechanical sound categories (Fig. 6*A*–*D*). The analysis revealed no significant correlations between soundscape pleasantness or eventfulness and the SCR amplitude across the two sound categories.

### Pleasantness and eventfulness correlate with SCR rise-time

To investigate whether there were associations between the SCR rise-time and the soundscape pleasantness and eventfulness, we correlated (Spearman) these factors across nature and mechanical sounds (Fig. 6). Significant positive correlations between the SCR rise-time and the soundscape pleasantness were observed in nature (*r* = 0.52; *p* = 0.008; *p-*adj = 0.034; Fig. 6*E*) and mechanical sounds (*r* = 0.4; *p* = 0.048; *p-*adj = 0.048; Fig. 6*G*), suggesting that the sounds with higher pleasantness prompted longer SCR rise-time across both sound categories. Inversely, we found significant negative moderate correlations between the SCR rise-time and the soundscape eventfulness in nature (*r* = −0.46; *p* = 0.028; *p-*adj = 0.034; Fig. 6*F*) and mechanical sounds (*r* = −0.44; *p* = 0.025; *p-*adj = 0.034; Fig. 6*H*) which evidenced that sounds with higher levels of eventfulness tend to elicit shorter SCR rise-time in nature and mechanical sounds. When controlling for eventfulness, partial correlation analyses revealed that the associations between SCR rise-time and pleasantness were no longer significant in both nature (*r* = 0.4; *p* = 0.053; *p-*adj = 0.106) and mechanical sounds (*r* = 0.24; *p* = 0.267; *p-*adj = 0.267). Similarly, the correlations between SCR rise-time and eventfulness were nonsignificant when controlling for pleasantness (nature, *r* = −0.31; *p* = 0.134; *p-*adj = 0.161; mechanical, *r* = −0.31; *p* = 0.13; *p-*adj = 0.161). This is likely due to the opposing effects of pleasantness and eventfulness.

All correlations were exploratory and not part of an a-priori analysis plan. To account for multiple comparisons, we applied FDR correction and report both adjusted and unadjusted *p*-values (Figs. 5*C*,*D*, 6).

## Discussion

This study explored how loudness influences variance in soundscape pleasantness, eventfulness, and their associated SCR. It also investigated the distinct effects of natural versus mechanical sound categories on these affective and physiological measures. Findings reveal that loudness significantly modulates SCR, with time series analysis showing that SCR differentiates physiological arousal between loud natural and mechanical sounds. No significant correlation emerged between SCR and subjective ratings of pleasantness or eventfulness; however, SCR rise-time showed significant associations with both pleasantness and eventfulness across sound categories. These results suggest that physiological arousal in response to soundscape is primarily driven by acoustic intensity, whereas perceptual qualities are more strongly tied to the nature of the sound source.

### SCR is influenced by loudness

The results demonstrated that SCR increased significantly as the loudness levels increased for both sound categories. Evidence has indicated that the SCR is highly sensitive to sound intensity (Grings et al., 1969; Björk, 1986; Bradley et al., 2000a; Cacioppo et al., 2007; Dawson et al., 2007; Bach et al., 2008, 2009; Boucsein, 2012; Bach, 2014; Gatti et al., 2018). For instance, Czepiel et al. (2021) found that louder musical tones elicited greater SCR, while Bari et al. (2024) reported that brief noise bursts (5 s) at higher intensity levels significantly increased SCR. Ellermeier and colleagues further corroborated these findings by demonstrating a direct correlation between the SPL of environmental noise (e.g., vehicles passing by) and SCR magnitude (Ellermeier et al., 2020). Additionally, studies on ventilation equipment noise presented at levels ranging from 35 to 75 dB(A) SPL in 2 min blocks have shown that increasing auditory intensity results in greater electrodermal activity (Alvar and Francis, 2024).

Empirical data, in this regard, show that rising sound intensity can be potentially perceived as salient warning cues or even a looming threat (Burow et al., 2005; Bach et al., 2008; LeDoux, 2022) and thereby prompts the recruitment of attentional and physiological resources to elicit adaptive responses. In a more precise manner, the effect of sound intensity on neural activity in the amygdala has been noted (Bach et al., 2008). The amygdala, in turn, plays a regulatory role in SNS activity through its projection to the hypothalamus. Importantly, the innervation of sweat glands is predominantly cholinergic and sympathetic in nature. This involves postganglionic fibers that originate from the sympathetic chain, as detailed by Shields et al. (1987). This implies a compelling causal relationship between the intensity of sound and the episodic bursts of sympathetic nerve activity that eventually result in the manifestation of the SCR.

### Skin conductance is modulated by sound category only when it reaches high loudness levels

Our results indicate that SCR is predominantly modulated by stimulus loudness/intensity rather than the categorical nature of the auditory input. Studies using controlled auditory paradigms have demonstrated that sounds from distinct categories such as nature (e.g., rain, wind), human (e.g., vocalizations), and mechanical (e.g., alarms, machinery) elicit comparable SCR magnitudes when matched for intensity (Bradley et al., 2000a; Hedblom et al., 2017; Kong and Han, 2024). This finding is consistent with the established role of the SNS, mediated through its connection with the salience network (Xia et al., 2017; Sturm et al., 2018; Seeley, 2019), in responding primarily to the physical salience of sensory input as opposed to categorical or affective interpretation (Seeley, 2019). Moreover, neurophysiological models suggest that the amygdala (Morris et al., 2001; Cheng et al., 2007) and brainstem structures (Cacioppo et al., 2007; Dawson et al., 2007), via two relatively independent pathways leading to SCR generation (Edelberg, 1972; Boucsein, 2012), play a crucial role in mediating autonomic responses to auditory stimuli based on their acoustic properties rather than their semantic content (Dawson et al., 2007). The relative invariance of SCR to sound category highlights its role as a nonspecific index of physiological arousal, mainly governed by low-level acoustic features rather than higher-order perceptual processing.

This observation, however, did not persist when contrasting the time series data of loud nature sounds relative to mechanical sounds, as time series analysis is sensitive to transient and rapidly fluctuating responses over time. This issue was discussed by Bach and Friston (2013), shedding light on the limitations of traditional operational approaches in SCR analysis which may overlook the temporal structure of physiological responses. Instead, they advocate for model-based methods that more accurately capture the underlying generative processes driving SCR dynamics. The differences in SCR between these conditions could be due to the dynamic interaction between loudness and frequency in real-world auditory perception, wherein changes in one parameter can affect the salience of the other (Neuhoff et al., 1999). Increasing loudness can enhance the prominence of specific frequency components and may even induce pitch shifts (Neuhoff et al., 2002). At higher loudness levels, acoustic properties inherent to mechanical sounds, such as their higher spectral content (Yang and Kang, 2013), may become more pronounced. These psychoacoustic features are particularly salient and attention-grabbing, demanding greater cognitive resources and prioritizing threat detection. Consequently, mechanical sounds may elicit heightened autonomic arousal at higher levels, leading to stronger physiological responses (Storbeck and Clore, 2008).

### Soundscape pleasantness and eventfulness are mediated by sound category irrespective of loudness

The extant literature validates mechanical sounds are typically regarded as unpleasant, whereas nature sounds tend to elicit pleasantness. Each sound category possesses distinct acoustic characteristics (e.g., decibel level/intensity in mechanical sounds; Nooralahiyan et al., 1998; Quaranta and Dimino, 2007) such that one, more than one, or the interaction of these inherent acoustic characteristics modulates the pleasantness and eventfulness of the soundscape. Considering that loudness is an acoustic property strongly tied to unpleasantness (Mitchell et al., 2021), we controlled for loudness to examine the extent to which loudness, as an inherent acoustic feature, contributes to the soundscape pleasantness and eventfulness of nature versus mechanical sounds. The findings confirmed the previous work, evidencing that soundscape pleasantness evoked by nature sounds was significantly higher relative to mechanical sounds, even when matched for the loudness level (e.g., 20 sones). In contrast, mechanical sounds were perceived as more eventful than nature sounds, even at equal loudness levels.

The present investigation is consistent with past research positing that nature sound scenarios are generally rated as more pleasant and less eventful compared with their mechanical counterparts. This observation may be attributed to inherent acoustic features that are typically present in nature sounds including more energy at low frequencies than at high (Voss and Clarke, 1978) and slow temporal modulations (Attias and Schreiner, 1997; Singh and Theunissen, 2003). Sounds containing elevated levels of energy at higher frequencies elicit aversive responses in listeners, while those with lower-frequency content are more pleasant (Patchett, 1979; Kumar et al., 2008). Additionally, temporal modulation of sounds within the roughness range (30–150 Hz) has been shown to induce unpleasantness, aversion, and defense reactions (Arnal et al., 2019; Taffou et al., 2021). In this regard, mechanical sounds may predominantly possess inherent acoustic properties (e.g., roughness, sharpness, and loudness) that are tied to disgust, aversion, excitability, unpleasantness, and perceptual arousal. These findings imply that the perceptual attributes of the soundscape related to nature and mechanical sounds are unlikely to be accounted for by mere decibel level/intensity. Future research is warranted to tease apart the degree of contribution of other psychoacoustic features, such as spectral content, within each sound category.

### Perceptual attributes demonstrate concordance with SCR rise-time

We observed no association between mean SCR (across the sound presentation epoch) and soundscape pleasantness and eventfulness, where eventfulness refers to the perceptual intensity and temporal dynamism of the auditory experience. We then explored SCR rise-time and velocity, both of which provide temporally sensitive indices of autonomic arousal (Dawson et al., 2007; Boucsein, 2012; Jindrová et al., 2020). The SCR rise-time is widely acknowledged to be inversely proportional to the magnitude of the gate current [most SCR require a gate current of 0.1–50 mA (milliamperes) to fire] and its buildup rate (Dawson et al., 2007). While SCR rise-time was significantly correlated with perceptual attributes ratings, it did not vary significantly across sound categories or loudness levels. In contrast, SCR velocity was modulated by loudness in mechanical sounds and by sound category at high loudness. We exclusively prioritized SCR rise-time for these exploratory correlations because it is a more established and interpretable index in the SCR literature for quantifying stimulus salience and arousal latency (Dawson et al., 2007; Boucsein, 2012).

SCR velocity, a less commonly used SCR metric (Lim et al., 2003), and SCR rise-time respectively represent the speed and duration required for the skin electrical conductance to elevate from the baseline to its peak level in response to a stimulus. These parameters are indices of the speed at which the SNS reacts. An SCR with a faster velocity and shorter rise-time signifies more rapid SNS activation, while a slower velocity and longer rise-time indicate slower activation. Nonetheless, factors such as the intensity of the stimulus may impact these indices. The SNS plays a key role in the “fight or flight” response, which is the body automatic reaction to perceived threat or danger. In such circumstances, the SNS must respond promptly to prepare the organism for a defensive reaction. Consequently, stimuli that possess greater perilous implications for the organism stimulate a more pronounced “fight or flight” response, as demonstrated by a faster SCR velocity and shorter rise-time, which enables the organism to effectively prepare for an evasive response to ward off the perceived danger (Storbeck and Clore, 2008).

These results suggest that while SCR velocity is likely to represent stimulus-driven automatic shifts linked to physical sound properties, SCR rise-time may capture early-stage affective appraisal of soundscapes. The latter is consistent with previous findings in the visual domain, where shorter SCR rise-time, characterized by a steeper slope, was observed for emotionally intense or unpleasant stimuli (Jindrová et al., 2020). Though rise-time has not extensively been studied in psychophysiology across domains, it may offer potential an informative marker of levels of arousal (Dawson et al., 2007; Boucsein, 2012; Jindrová et al., 2020), especially given its associations with both pleasantness and eventfulness. It should be noted that these findings are exploratory and were not part of an a-priori analysis plan. While the observed dissociation between SCR rise-time and subjective ratings, as well as the associations between SCR velocity in response to natural versus mechanical sounds at high loudness levels, require further empirical validation, they nonetheless contribute to our understanding of soundscape perception by highlighting distinct SCR components of the autonomic response. These findings may inform future research on the temporal dynamics of sympathetic engagement in auditory processing, particularly in relation to the salience network (Seeley et al., 2007; Touroutoglou et al., 2012; Xia et al., 2017).

### Multisensory modulation of soundscape perception

While our study focused exclusively on auditory stimuli, it is important to note that real-world soundscapes are typically experienced in conjunction with other sensory information (Vroomen and de Gelder, 2000; Frassinetti et al., 2002; Stefanics et al., 2005; Recanzone, 2009; Calcagno et al., 2012; Liu et al., 2014; Jeon and Jo, 2020; Xu and Wu, 2021; Zhang et al., 2025). In this regard, vision has a powerful modulatory effect on how sounds are perceived, particularly in terms of affective and qualitative appraisals such as pleasantness, arousal, and restorativeness (Li and Liu, 2024). Studies have shown that the visual context in which a sound is experienced can either amplify or attenuate its affective impact (Opoku-Baah et al., 2021). For example, the same urban noise may be rated as significantly more pleasant or less annoying when paired with visual scenes of natural environments, such as parks or greenery, as opposed to built or traffic-heavy settings (Viollon et al., 2002; Hong and Jeon, 2014). Similarly, auditory and olfactory modalities have been shown to interact, particularly when the stimuli are affectively congruent (Seo and Hummel, 2011). These effects are attributed to cross-modal integration processes, wherein other sensory cues provide contextual information that influences the interpretation of concurrent auditory input (Stefanics et al., 2005; Petro et al., 2017; Zhou et al., 2019; Opoku-Baah et al., 2021; Gao et al., 2023).

## Conclusion

This study presents empirical evidence for the impact of loudness and sound category on soundscape perceptual and physiological attributes. Key findings indicate that while loudness levels modulate SCR, with SCR increasing as loudness rises, pleasantness and eventfulness remain unaffected. Conversely, the sound category (nature and mechanical) influences the pleasantness and eventfulness of the soundscape. The change in SCR does not correspond to the variance in pleasantness and eventfulness; however, SCR rise-time, which is inversely proportional to SCR amplitude, is associated with pleasantness and eventfulness. Collectively, this study provides validated insights into the acoustic properties that impact the affective dimensions of sound perception and the associated physiological substrates. They are of practical relevance for soundscape researchers, auditory neuroscientists, audiologists, and sound designers, who can use this knowledge to create healthier and more optimal acoustic environments by carefully considering relevant acoustic properties.

## Synthesis

Reviewing Editor: James Howard, Brandeis University Decisions are customarily a result of the Reviewing Editor and the peer reviewers coming together and discussing their recommendations until a consensus is reached. When revisions are invited, a fact-based synthesis statement explaining their decision and outlining what is needed to prepare a revision will be listed below. The following reviewer(s) agreed to reveal their identity: NONE.

In this paper the authors examined subject rating responses and skin conductance responses to multiple exemplars of two categories of soundscapes: nature sounds and mechanical sounds. They found that mechanical sounds were rated as more pleasant and more eventful than nature sounds. In terms of SCR, they found higher amplitude responses for louder sounds, and correlations between SCR rise time and subjective ratings of the sounds. This is a solid study that uses methodologically sound techniques to explore responses to these soundscapes. The findings confirm previous studies concerning arousal responses related to loudness and subjective pleasantness. Given the relatively small sample size of the study the exploratory findings may be of limited impact to the field - however, the results could be of interest to auditory and perceptual researchers. The detailed comments and suggestions of the reviewers are as follows:

1. Lines 148-150: "However, since the auditory stimuli in the IADS - E were short (1.5 s), we adopted the same stimuli (with the same label such as jackhammer in the mechanical sound category) with longer duration from other sources."

This statement is slightly misleading. The authors appear to be using a standardized database of sounds to identify stimuli that fall within desired quantiles of measured arousal and valence. They are then finding sounds with the same semantic label as those database stimuli in other sources. These are not the "same stimuli," and without independent verification of the arousal and valence of the chosen 15-s sound clips, it is not possible to know whether they also fall within the presumed quantiles of arousal and valence. The subjective ratings acquired in the active listening task here may speak to this, but it is not accurate to describe them as the same stimuli. This aspect of the methods should be more clearly described, and the authors should acknowledge that the stimuli used in the current experiment do not come from the database.

2. Lines 151-153: "The objective of this experiment was to employ a diverse corpus of sounds that effectively encompassed each category of nature and mechanical sounds and were widely distributed across the valence and arousal spectra."

Having 3 scenarios within the 2 sound categories does not constitute a diverse corpus of sounds. In fact, it is quite selective relative to vast array of possible nature and mechanical sounds that could have been used here. The language used to describe selection of the stimuli used here should be modified to reflect the selectivity of the process and to acknowledge as a limitation that the specificity of these particular stimuli may indeed limit the generalizability of the findings.

3. It should be made more clear in the Methods section that SCR was only recorded during the passive listening task, and therefore all analyses of those signals come from that task.

4. Line 185: There is a reference to Figure 1 here, but the information presented in Figure 1 does not really match up with the text. Figure 1A should be referenced in the context of the results of the discrimination task, and Figure 1B should be referenced when describing the quantification of the loudness of sound stimuli (or something like that). In general, throughout the manuscript care should be taken to reference specific figure panels with the information they portray.

5. Related to the previous point, a schematic figure showing aspects of the task design and timing would be helpful to include in Figure 1 to give the reader a better visual sense of what participants were doing in this experiment.

6. In Figure 1B, the Low, Medium, and High sounds are all labeled as 10 sone.

7. The panels in Figure 2 are labeled A, B, and D (C is missing), and are not referenced accurately in the text. The plot in panel A is somewhat redundant in panels B and D, as it is just data averaged over those conditions. Given that the main analysis was a 2-way ANOVA with loudness and category as factors, it seems appropriate to have one figure panel that incorporates all the data presented in panels 2B and 2D. Alternatively just leave them as separate panels without duplicating the silence condition.

8. The SCR responses for the "silence" condition appear to be duplicated in panels 2B and 2D. If this is the case, these data should be presented once and not duplicated.

9. In Figure 3 loudest sound is sometimes referred to as "High" and sometimes as "Loud." Please be consistent throughout the figures and the text of the manuscript in the labeling of the conditions.

10. In Figure 5A and B it's a bit visually confusing to include thick vertical rectangles for SD, thick rectangles for quartiles, and box markers and the kernel densities for percentiles (as described in the figure legend) all on the same figure. I actually don't see the rectangles marking SD - maybe they're not actually there? At any rate it is too much visual information to make sense of so we suggest removing the kernel densities and perhaps only showing data points, mean, and SD. The extent of the quartiles and percentiles is not particularly helpful to tell the story beyond those measures and the cited statistics.

11. The motivation for testing correlations between pleasantness/eventfulness and SCR rise-time is questionable. There was no effect of condition on SCR rise-time, but there were some effects of condition on SCR velocity. The correlations between SCR rise-time and subjective ratings are thus entirely exploratory analysis, and as such the resulting correlations should be corrected for multiple comparisons. In addition, a better justification should be made, either in the Results or Discussion section, for why these correlations were explored when SCR velocity was not, and what these findings really add to the overall results.

12. Related to the previous point, it seems in the Discussion section that SCR velocity and rise-time refer to the same thing - for example on lines 556-558 a claim is made that sound category has a significant impact on both. This is inconsistent with what is presented in the results, which state no effect of sound category on SCR rise-time. The authors should take care to discuss the results accurately and to disambiguate the treatment of SCR velocity and SCR rise-time throughout the manuscript.

13. Since a soundscape is not equivalent to a sound source itself, most existing research also incorporates visual stimuli in their analyses. It would be valuable to at least briefly discuss the relationship between auditory and other sensory modalities in the manuscript.

## Author Response

Dear Dr Howard, Thank you for the opportunity to revise our manuscript. We appreciate the detailed and constructive feedback, which has helped us improve the quality of our paper.

We have addressed each comment in our revision. All changes are described below and highlighted in green in the manuscript.

1. Lines 148-150: "However, since the auditory stimuli in the IADS - E were short (1.5 s), we adopted the same stimuli (with the same label such as jackhammer in the mechanical sound category) with longer duration from other sources." This statement is slightly misleading. The authors appear to be using a standardized database of sounds to identify stimuli that fall within desired quantiles of measured arousal and valence. They are then finding sounds with the same semantic label as those database stimuli in other sources. These are not the "same stimuli," and without independent verification of the arousal and valence of the chosen 15-s sound clips, it is not possible to know whether they also fall within the presumed quantiles of arousal and valence. The subjective ratings acquired in the active listening task here may speak to this, but it is not accurate to describe them as the same stimuli. This aspect of the methods should be more clearly described, and the authors should acknowledge that the stimuli used in the current experiment do not come from the database.

Response: Thank you. We agree with this point and have now clarified it in the main manuscript (Page 7, Line 14). "Since the auditory stimuli in the IADS-E were short (1.5 s), we selected task stimuli with the same labels (e.g., jackhammer in the mechanical sound category) but with longer durations from other sources. Although these sounds shared the same semantic labels as those in the IADS-E (e.g., jackhammer), they cannot be assumed to match its normative arousal and valence ratings. Instead, the IADS-E ratings were only used to guide the selection of sound categories and exemplars expected to span the affective space." 2. Lines 151-153: "The objective of this experiment was to employ a diverse corpus of sounds that effectively encompassed each category of nature and mechanical sounds and were widely distributed across the valence and arousal spectra." Having 3 scenarios within the 2 sound categories does not constitute a diverse corpus of sounds. In fact, it is quite selective relative to vast array of possible nature and mechanical sounds that could have been used here. The language used to describe selection of the stimuli used here should be modified to reflect the selectivity of the process and to acknowledge as a limitation that the specificity of these particular stimuli may indeed limit the generalizability of the findings.

Response: Thank you for this comment and we agree with this important point. Thus, we have addressed this in the manuscript (Page 7, Line 21). "While the selected stimuli were intended to represent a range of affective values within each category, we acknowledge that the six scenarios (three per category, each with four exemplars) constitutes a relatively narrow and selective sample of possible nature and mechanical sounds. As such, this specificity may limit the generalizability of our findings to other soundscapes within these broader categories. Our intention was not to exhaustively sample the category space, but rather to use a controlled subset of representative sounds to examine affective and physiological responses across distinct semantic and emotional profiles." 3. It should be made more clear in the Methods section that SCR was only recorded during the passive listening task, and therefore all analyses of those signals come from that task.

Response: This point was already noted on Page 11 (highlighted in yellow). We have also now made it more explicit in additional places including in the schematic figure (Figure 2) and the relevant subheading: "Passive listening task (SCR recorded)" (Page 10, Line 1) "Active listening task (Perceptual attributes ratings)" (Page 11, Line 7) "... and no SCR was recorded during this task." (Page 11, Line 17) 4. Line 185: There is a reference to Figure 1 here, but the information presented in Figure 1 does not really match up with the text. Figure 1A should be referenced in the context of the results of the discrimination task, and Figure 1B should be referenced when describing the quantification of the loudness of sound stimuli (or something like that). In general, throughout the manuscript care should be taken to reference specific figure panels with the information they portray.

Response: Thank you for this comment and your careful reading. We have now properly referenced Figure 1 in three relevant locations in the manuscript: (1) Page 8, Line 13, where we describe the normalization of stimuli at three loudness levels; (2) Page 9, Line 12, where we report the loudness discrimination task; and (3) Page 16, Line 21 and 22, where we report comparisons of averaged SCR across sound categories. All figure references have been double-checked throughout the manuscript for consistency.

5. Related to the previous point, a schematic figure showing aspects of the task design and timing would be helpful to include in Figure 1 to give the reader a better visual sense of what participants were doing in this experiment.

Response: We have now added a schematic figure illustrating the task design and timing (Figure_2.tiff), along with its caption at the end of the manuscript.

6. In Figure 1B, the Low, Medium, and High sounds are all labeled as 10 sone.

Response: This has been modified in Figure 1A.

7. The panels in Figure 2 are labeled A, B, and D (C is missing), and are not referenced accurately in the text. The plot in panel A is somewhat redundant in panels B and D, as it is just data averaged over those conditions. Given that the main analysis was a 2-way ANOVA with loudness and category as factors, it seems appropriate to have one figure panel that incorporates all the data presented in panels 2B and 2D. Alternatively just leave them as separate panels without duplicating the silence condition.

8. The SCR responses for the "silence" condition appear to be duplicated in panels 2B and 2D. If this is the case, these data should be presented once and not duplicated.

Response (Address both comments 7 and 8): Thank you for these comments. To address reviewers' comments 7 and 8, we have combined the previous three panels from Figure 2 (old version) into a single panel that presents averaged SCRs in response to both natural and mechanical sounds across the three loudness levels. Only one silence condition (bar) is included to reduce redundancy. This revised panel has been incorporated into Figure 1 (new version) as Panel C (See Figure 1 attached as response to comment 6). The caption has been modified accordingly.

9. In Figure 3 loudest sound is sometimes referred to as "High" and sometimes as "Loud." Please be consistent throughout the figures and the text of the manuscript in the labeling of the conditions.

Response: This has been addressed in the revised Figure 3 and updated consistently throughout the manuscript and any related figures, where applicable.

10. In Figure 5A and B it's a bit visually confusing to include thick vertical rectangles for SD, thick rectangles for quartiles, and box markers and the kernel densities for percentiles (as described in the figure legend) all on the same figure. I actually don't see the rectangles marking SD - maybe they're not actually there? At any rate it is too much visual information to make sense of so we suggest removing the kernel densities and perhaps only showing data points, mean, and SD. The extent of the quartiles and percentiles is not particularly helpful to tell the story beyond those measures and the cited statistics.

Response: This has been revised in Figure 5 by including vertical rectangles for standard deviations, thick lines representing means, and individual data points. The figure caption has been updated accordingly.

11. The motivation for testing correlations between pleasantness/eventfulness and SCR rise-time is questionable. There was no effect of condition on SCR rise-time, but there were some effects of condition on SCR velocity. The correlations between SCR rise-time and subjective ratings are thus entirely exploratory analysis, and as such the resulting correlations should be corrected for multiple comparisons. In addition, a better justification should be made, either in the Results or Discussion section, for why these correlations were explored when SCR velocity was not, and what these findings really add to the overall results.

Response: Thank you for raising this important point. We agree that the analyses involving correlations between SCR rise-time and perceptual ratings were exploratory in nature. In response to this comment, we have taken the following steps:

1 - We now clearly label these correlation analyses as exploratory in the Results section (Page 22, Line 1, page 26, line 12 as well as the relevant captions). We have added a paragraph to the Discussion that contextualizes these exploratory findings more cautiously, emphasizing their tentative nature and suggesting that future work is needed to replicate and further examine these associations and we explicitly state that they were not hypothesized a priori (Page 27, Line 10). "It should be noted that these findings are exploratory and were not part of an a priori analysis plan. While the observed dissociation between SCR rise time and subjective ratings, as well as the associations between SCR velocity in response to natural versus mechanical sounds at high loudness levels, require further empirical validation, they nonetheless contribute to our understanding of soundscape perception by highlighting distinct SCR components of the autonomic response. These findings may inform future research on the temporal dynamics of sympathetic engagement in auditory processing, particularly in relation to the salience network (Seeley et al., 2007; Touroutoglou et al., 2012; Xia et al., 2017)." 2- We have corrected all the correlations for multiple comparisons using the False Discovery Rate (FDR) approach and report the adjusted p-values alongside the original p-values (Page 22, Line 6; Figure 5 and 6 and the corresponding legends also were updated).

3- While we did observe condition effects on SCR velocity, we prioritized SCR rise-time for these exploratory correlations because it is a more established and interpretable index in the SCR literature for quantifying stimulus salience and arousal latency (e.g., Dawson et al., 2007; Boucsein, 2012). Nevertheless, we now acknowledge this rationale explicitly in the Discussion (Page 26, Line 18) and clarify the distinction between rise-time and velocity. "We exclusively prioritized SCR rise-time for these exploratory correlations because it is a more established and interpretable index in the SCR literature for quantifying stimulus salience and arousal latency (Boucsein, 2012; Dawson et al., 2007)." 12. Related to the previous point, it seems in the Discussion section that SCR velocity and rise-time refer to the same thing - for example on lines 556-558 a claim is made that sound category has a significant impact on both. This is inconsistent with what is presented in the results, which state no effect of sound category on SCR rise-time. The authors should take care to discuss the results accurately and to disambiguate the treatment of SCR velocity and SCR rise-time throughout the manuscript.

Response: Thank you for pointing out this inconsistency. We have revised the relevant sentence in the Discussion to clarify that only SCR velocity was significantly affected by sound category and loudness. We have also disambiguated the roles of SCR velocity and rise-time throughout the manuscript to ensure accuracy.

We also replaced Lines 556 - 558 with the following sentence: "We observed no association between mean SCR (across the sound presentation epoch) and soundscape pleasantness and eventfulness, where eventfulness refers to the perceptual intensity and temporal dynamism of the auditory experience. We then explored SCR rise-time and velocity, both of which provide temporally sensitive indices of autonomic arousal (Boucsein, 2012; Dawson et al., 2007; Jindrová et al., 2020). The SCR rise-time is widely acknowledged to be inversely proportional to the magnitude of the gate current, (most SCR require a gate current of 0.1 - 50 mA (milliamp) to fire), and its build-up rate (Dawson et al., 2007). While SCR rise-time was significantly correlated with perceptual attributes ratings, it did not vary significantly across sound categories or loudness levels. In contrast, SCR velocity was modulated by loudness in mechanical sounds and by sound category at high loudness" (Page 26, Line 9).

13. Since a soundscape is not equivalent to a sound source itself, most existing research also incorporates visual stimuli in their analyses. It would be valuable to at least briefly discuss the relationship between auditory and other sensory modalities in the manuscript.

Response: Thank you for this helpful suggestion. We have added a brief discussion of the relationship between auditory and other sensory modalities and highlighted the importance of other sensory perception in shaping soundscape. This addition appears in the Discussion section (Page 27, Line 14). "Multisensory modulation of soundscape perception While our study focused exclusively on auditory stimuli, it is important to note that real-world soundscapes are typically experienced in conjunction with other sensory information (Calcagno et al., 2012; Frassinetti et al., 2002; Jeon et al., 2020; Liu et al., 2014; Recanzone, 2009; Stefanics et al., 2005; Vroomen et al., 2000; Xu et al., 2021; Zhang et al., 2025). In this regard, vision has a powerful modulatory effect on how sounds are perceived, particularly in terms of affective and qualitative appraisals such as pleasantness, arousal, and restorativeness (Li et al., 2024). Studies have shown that the visual context in which a sound is experienced can either amplify or attenuate its affective impact (Opoku-Baah et al., 2021). For example, the same urban noise may be rated as significantly more pleasant or less annoying when paired with visual scenes of natural environments, such as parks or greenery, as opposed to built or traffic-heavy settings (Hong et al., 2014; Viollon et al., 2002). Similarly, auditory and olfactory modalities have been shown to interact, particularly when the stimuli are affectively congruent (Seo et al., 2011). These effects are attributed to cross-modal integration processes, wherein other sensory cues provide contextual information that influences the interpretation of concurrent auditory input (Gao et al., 2023; Opoku-Baah et al., 2021; Petro et al., 2017; Stefanics et al., 2005; Zhou et al., 2019)."
